# A belly full of jelly? DNA metabarcoding shows evidence for gelatinous zooplankton predation by several fish species in Greenland waters

**DOI:** 10.1098/rsos.240797

**Published:** 2024-08-14

**Authors:** Annkathrin Dischereit, Julia Katharina Throm, Karl Michael Werner, Stefan Neuhaus, Charlotte Havermans

**Affiliations:** ^1^ HYIG ARJEL, Benthic Ecology, Alfred Wegener Institute Helmholtz Centre for Polar and Marine Research, Bremerhaven, Germany; ^2^ Marine Zoology, BreMarE—Bremen Marine Ecology, Fachbereich 2, Universität Bremen, Bremen 28334, Germany; ^3^ Thünen Institute of Sea Fisheries, Bremerhaven, Germany; ^4^ Data Division, Alfred Wegener Institute Helmholtz Centre for Polar and Marine Research, Bremerhaven, Germany

**Keywords:** Greenland waters, gelatinous zooplankton, diet composition, DNA metabarcoding, fish assemblages

## Abstract

The waters of Greenland harbour a high species richness and biomass of gelatinous zooplankton (GZP); however, their role in the diet of the many fish species, including commercially exploited species, has not yet been verified. Traditionally, GZP was considered to be a trophic dead end, i.e. with a limited contribution as prey for higher trophic levels. We applied DNA metabarcoding of two gene fragments (COI, 18S V1–V2) to the stomach contents of seven pelagic and demersal fish species in Greenland waters, to identify their prey composition as well as the occurrence of GZP predation. We detected GZP DNA reads in the stomachs of all investigated fish species, with frequency of occurrences ranging from 12.5% (for *Melanogrammus aeglefinus*) to 50% (for *Argentina silus*). GZP predation had not yet been reported for several of these species. GZP were found to majorly contribute to the diet of *A. silus* and *Anarhichas denticulatus*, particularly, the siphonophore *Nanomia cara* and the scyphozoan *Atolla* were of a high importance as prey, respectively. The use of multiple genetic markers enabled us to detect a total of 59 GZP taxa in the fish stomachs with several GZP species being detected only by one of the markers.

## 1. Introduction

Gelatinous zooplankton (hereafter ‘GZP’) refers to a number of phylogenetically very different taxa: cnidarian medusae (including hydrozoans and scyphozoans), ctenophores and pelagic tunicates (e.g. salps, appendicularians). These groups have in common that they have fragile, watery bodies that are often transparent. They occur in all oceans, from polar to tropical regions, and occupy all depth zones, from the epipelagic zone to the deep sea. Generally, GZP are reputed to be climate-change winners, with a number of temperate species reported to increase with warmer waters [1–3] and poleward range shifts have been observed for several boreal species [1,2]. Climate change is generally driving the World’s oceans towards a warmer, fresher and more acidic state [4,5]. These new conditions, including other anthropogenic changes such as eutrophication, pollution, habitat modifications and overfishing [6], generate ‘simplified’ ecosystems, where GZP can thrive. In some cases, they even induce regime shifts with highly productive, fish-dominated food webs being replaced by jelly-dominated, less productive ones [7,8].

The ‘Ocean jellification’ paradigm is supported by a number of pelagic datasets showing an increase in GZP biomass or blooms in several marine ecosystems worldwide (e.g. Black Sea, East China Sea or the Northeast US continental shelf [9]) and/or by the growing records of negative impacts of GZP aggregations on human enterprises, including fisheries, tourism and clogging of water-intakes of power production systems [6,9,10]. Furthermore, GZP are in the position to outcompete fish in stressful environments or prevent fish stock recovery after overfishing (e.g. Irish Sea [11]) since they often compete with fish for the same food. Even though evidence is accumulating, this jellification paradigm is still under heavy debate [12,13], due to the critical scarcity of baseline data [14]. The combination of the typical boom-and-bust population dynamics of GZP, causing population fluctuations over different temporal scales [15–17] and the lack of reliable, long-term abundance datasets due to less effective sampling techniques (e.g. [18]) have blurred our view on their long-term, climate-change-driven, population trends.

Considering that GZP composes a large fraction of the pelagic biomass and may become even more central in several marine systems, their ecosystem impact as prey may similarly increase [19,20]. Traditionally, the contribution of GZP to the energy budgets of predators has been greatly underestimated, being considered as a ‘trophic dead end’ in the food web [19]. Their watery nature and delicate tissues are quickly digested in predators’ stomachs, which is why their contribution to predators’ diets is frequently overlooked using conventional (microscopy) stomach analyses. GZP was considered at the most a survival food for some fish species [21], and this perception has changed only in recent years, with the application of modern approaches (DNA metabarcoding, *in situ* observations). With these tools, GZP predation has been shown to be much more common than previously assumed, although the importance of GZP as prey in the pelagic trophic webs is still difficult to quantify [14]. Many fishes, several of which are of commercial importance [22–24], birds [25,26], turtles [27,28] and cephalopods [29] routinely target GZP as part of their diet. Various other invertebrates like shrimps (*Pandalus borealis* [30]); and amphipods [31] feed on GZP, whereas several scavengers, including hagfish and different crustaceans, were found to feed on their sunken carcasses [32–34]. Despite their low energy density, the contribution of GZP to the energy budgets of predators may be more considerable than previously hypothesized, due to their rapid digestion, low capture costs, their availability in high numbers and a selective feeding on their energy-rich components [20]. This paradigm shift challenges our current understanding of pelagic ecosystem functioning, particularly in view of recent GZP increases, and hence deserves further validation for different marine ecosystems.

Southern Greenland waters are dominated by two main currents, the Central Irminger Current, composed of warmer and saline Atlantic waters, and the East Greenland Current, composed of colder and fresher Arctic waters [35,36]. These waters are important nursery grounds for different commercially exploited fish species, e.g. Atlantic cod (*Gadus morhua*), and as a fishing ground used by many different nations [37]. Of the 269 reported Greenlandic marine fish species, 80 species are known to spawn in Greenlandic waters [38,39]. The fish species occurring in Greenlandic waters can be divided into two main groups, according to their environmental affinities: boreal and arctic. While boreal species like herring, Atlantic cod, Atlantic halibut and haddock are associated with more temperate waters of the Irminger Current and the southern West Greenland Current, arctic species like polar cod are more abundant in colder waters of north and east Greenland [40,41]. Increased warming of the Irminger Current combined with changes in ice conditions have led to a regime shift in oceanographic and ecological conditions in southern Greenland waters, which manifested through changes in fish community composition [42–44]. In recent years, an influx of boreal species has been observed [44–47], and concomitantly, habitat loss and increased interspecific competition were observed and predicted for arctic species [48–51]. Such distribution shifts are likely to increase with ongoing environmental changes [37].

Greenland waters harbour a significantly higher species richness and biomass of GZP (i.e. in wet weight) than the neighbouring northeast Atlantic [52]. Cnidarian medusae are most species rich, while ctenophores, despite being represented by only a handful of species, can reach very high abundances in Greenland waters [38,53]. Similar to fish, community shifts in GZP are expected, with boreal species shifting northward and increasing in abundances in Arctic waters [2,9]. For the Fram Strait, the Atlantic gateway to the Arctic, an increase in GZP abundances was projected with increasing warming and ‘Atlantification’ [1]. Considering potential increases in GZP biomass in the near future, there is an urgent need to establish a better understanding of the current role of GZP in the southern Greenland marine food web. Hence, this study aims to investigate the prey spectrum and the role of GZP as prey for fish in Greenland waters. To do so, we apply a multi-locus DNA metabarcoding approach, allowing for a high taxonomic resolution in prey identification and facilitating the detection of otherwise easily overlooked GZP prey. We investigated the diet spectrum of the following seven species, caught during a demersal groundfish survey: Atlantic cod (*G. morhua*), golden redfish (*Sebastes norvegicus*), northern wolffish (*Anarhichas denticulatus*), spotted wolffish (*Anarhichas minor*), greater silver smelt (*Argentina silus*), American plaice (*Hippoglossoides platessoides*) and haddock (*Melanogrammus aeglefinus*). GZP predation has been demonstrated for some of these species in locations in the North Atlantic [54–56], but so far, this has not been tested yet in Greenland waters. We assessed whether the diet composition including GZP predation varies spatially and whether distinct GZP taxa act as dominant prey for the different fish species. Finally, by comparing the sequencing output between the targeted gene fragments of the mitochondrial gene cytochrome *c* oxidase I fragment (COI) and the nuclear 18S rDNA gene fragment (18S), we were able to obtain a comprehensive taxonomic coverage of the prey spectrum of the different fish species and unravel which GZP groups are more efficiently detected with each of these markers.

## 2. Material and methods

### 2.1. Sampling

**Figure 1.**
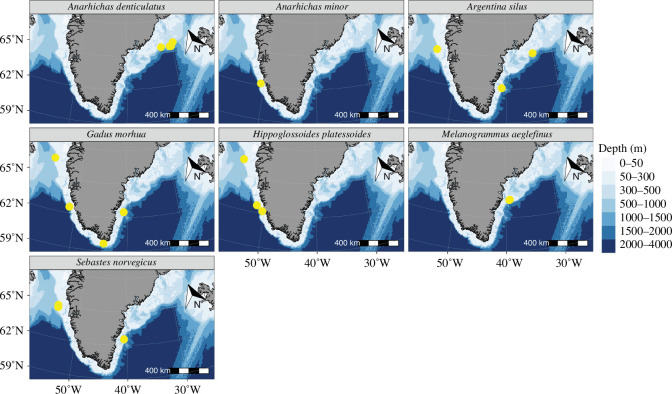
Map indicating the sampling locations for the different fish species investigated in this study. The individuals were collected in the framework of the ‘Greenland Groundfish Survey’ conducted by Thünen-Institute of Sea Fisheries on board of R/V Walther Herwig III in 2020.

Fish samples were collected as part of the annual ‘Greenland Groundfish Survey’ in southern, western and eastern Greenland waters, with a standardized 140-feet bottom trawl (Rockhopper) [57]. The survey was conducted by Thünen-Institute of Sea Fisheries on board R/V Walther Herwig III (WH440) in October and November 2020. Fish were opportunistically collected from trawls at different locations, depending on the size and nature of the catch. After the sorting of the catch, individuals were identified, weighed and standard length was measured. Seven fish species were targeted for the diet analysis: Atlantic cod (*G. morhua*), redfish (*S. norvegicus*), northern and spotted wolffish (*A. denticulatus, A. minor*), greater silver smelt (*A. silus*), American plaice (*H. platessoides*) and haddock (*M. aeglefinus*). A total of 132 individuals from 19 different locations were selected (figure 1). We removed the stomachs of larger individuals on board and kept them frozen at −20°C until further processing in the home laboratory. Smaller individuals, including all American plaice, were frozen whole and their stomachs were removed in the home laboratory.

### 2.2. DNA extractions

For each individual fish, the stomach was opened and its content photographed. The stomach content was isolated using sterilized pincers and its entire content was homogenized using a standard kitchen blender (KRUPS Perfectmix 9000 Mini-Standmixer) or an Ultra-Turrax (IKA). The latter was used when the volume of the stomach content was too small for the blender. Dissection instruments were sterilized using ethanol and a Bunsen burner, while knives and blender buckets were sterilized with diluted bleach and MilliQ water between each stomach isolation. DNA extractions were performed on a subsample of up to 25 mg of homogenized stomach contents. They were done using the DNeasy Blood & Tissue Kit (QIAGEN), following the manufacturer’s instructions and applying an elution volume of 100 µl. DNA concentration was measured using a Nanodrop ND-1000 spectrophotometer (Thermo Fisher Scientific). DNA extracts were stored at −20°C for further processing. A total of five extraction blanks were included, to account for cross-contamination between samples as well as general laboratory contamination.

### 2.3. Library preparation and sequencing

The library preparation and Illumina NovaSeq sequencing were carried out by AllGenetics & Biology SL (https://www.allgenetics.eu) following a two-step polymerase chain reaction (PCR) protocol (following [58]). To enable the detection of a broad range of metazoan prey in the stomachs, a multi-marker approach was applied, combining the ‘Leray’-fragment of the COI with the V1–V2 region of 18S rDNA. Both markers have been used in previous metabarcoding diet studies targeting metazoans and also showed evidence of GZP in the diet of different organisms [22,30,59].

The following highly degenerated Leray-XT primers were used to amplify a 313 bp long fragment of COI (Leray-fragment): the forward primer mlCOIintF-XT (5′-GGWACWRGWTGRACWITITAYCCYCC-3′) [59], and the reverse primer jgHCO2198 (5′-TAIACYTCIGGRTGICCRAARAAYCA-3′) [60]. Both primers included a binding side for the Illumina adapters, attached during the second PCR. The library preparation was carried out in a two-step PCR protocol. In the first step, the Leray-fragment was amplified. Depending on the sample quality, the first PCR was carried out in a total volume of 12.5 µl (most samples, for some DNA extract was diluted 1:10) or 25 µl for samples with low amplification success (eight samples). For the high-quality DNA samples, the PCR mix was prepared using 1.25 µl of DNA template, 0.5 µM of Leray-XT primers, 6.25 µl of Supreme NZYTaq 2 x Green Master Mix (NZYTech), and ultrapure water up to 12.5 µl. The PCR mix was incubated with an initial denaturation step at 95°C for 5 min, followed by 35 cycles of denaturing at 95°C for 30 s, annealing at 55°C for 45 s and extension at 72°C for 30 s, followed by a final extension step at 72°C for 10 min. The samples with poorer quality were amplified in a final volume of 25 µl, containing 1 µl of DNA template, 0.5 µM of Leray-XT primers, 12.5 µl of Supreme NZYTaq 2 x Green Master Mix (NZYTech), CES 1X and ultrapure water up to 25 µl. Additionally, for these samples, the annealing temperature was reduced from 55 to 54.7°C, while the remaining PCR conditions remained the same.

To amplify the approximately 356 bp long fragment within the variable region V1–V2 of the 18S rDNA gene (18S), we used the forward primer SSUF04 (5′-GCTTGTCTCAAAGATTAAGCC-3′) [61] and the reverse primer SSURmod (5′-CCTGCTGCCTTCCTTRGA-3′) [62]. The first PCR was conducted in a total volume of 12.5 µl, containing 1.25 µl of DNA template, 0.5 µM of the aforementioned primers, 3.13 µl of Supreme NZYTaq 2 x Green Master Mix (NZYTech) and ultrapure water up to 12.5 µl. The PCR conditions were set to the following steps: initial denaturation at 95°C for 5 min, followed by 35 cycles of 95°C for 30 s, 49.7°C for 45 s and 72°C for 45 s, followed by a final extension step at 72°C for 7 min. In the second PCR, oligonucleotide indices linked to Illumina universal adapters were attached to the amplified fragments from PCR1. This allows the pooling of several samples for sequencing. The PCR conditions for this second PCR were similar to the conditions in the respective first PCRs. However, in the PCR2, only five cycles were conducted, with a different annealing temperature of 60°C.

PCR negative controls (ultrapure water) were included at all PCR steps. Electrophoresis was run on an 2% agarose gel stained with GreenSafe (NZYTech) and visualized using UV imaging to check library fragment size. Libraries were purified using the Mag-Bind RXNPure Plus magnetic beads (Omega Biotek). The final libraries were quantified with a Qubit and the dsDNA HS Assay (Thermo Fisher Scientific) and were then pooled equimolar. The final pool was sequenced on a fraction of an Illumina NovaSeq paired-end 250 bp run.

### 2.4. Bioinformatics and data refinement

Due to the different properties of the two genetic markers used in this study, we applied distinct bioinformatic pipelines, which differed in their approach based on denoising versus clustering. As COI represents a fast-evolving mitochondrial marker, solely denoising would not be appropriate, as species-level diversity for such markers is measured in molecular operational taxonomic units (MOTUs) and not as exact sequence variants (ESVs) [63]. For nuclear, ribosomal markers with low variability such as 18S, a higher correspondence between unique sequences and species can be expected and thus solely denoising is appropriate in this case [63]. For both datasets, sequencing data were demultiplexed and adapters were removed by the sequencing company.

For the bioinformatic analysis of the COI data, we followed the MJOLNIR pipeline (Metabarcoding Joining OBItools and Linkage Networks In R) (v. 3.0.0) (https://github.com/adriantich/MJOLNIR3) in R v. 4.0.4 with the default parameters set for the Leray-XT primers. Within this pipeline, we applied OBItools (v. 3.0) [64] for paired-end alignment (overlap between forward and reverse reads of approximately 113 bp ± 2 bp), primer removal, read length filtering (299−320 bp) and quality filtering using a minimum average Phred quality score of 30 per sequence. VSEARCH (v. 2.23) [65] was used to remove chimeric sequences from the dataset. To cluster the sequences into MOTUs which represent a proxy for species, the SWARM algorithm (v. 3.1.4) [66] was applied with a clustering distance of *d* = 13. The taxonomic assignment was done using ecotag [64] within OBItools against a custom reference database available on https://drive.google.com/drive/folders/1dVfZYCwoIK6D2V7adhF4xt85WdxzUys7, made available by the MJOLNIR3 provider. This reference database is composed of eukaryotic sequences obtained from public reference databases including NCBI GenBank and the Barcode of Life reference database (BOLD).

For the analysis of the 18S dataset, primers were removed using cutadapt (v. 2.8) [67]. We applied selected functions of the R package ‘DADA2’ v. 1.18.0 [68] to filter and trim (220 bp) the raw reads considering base quality scores, to conduct read denoising according to the ‘Divisive Amplicon Denoising Algorithm’ (DADA), to merge denoised paired-end reads into amplicon sequence variants (ASVs) and to detect and remove putative chimeric sequences. Furthermore, we used the dada2-implementation of the RDP Naive Bayesian Classifier [69] with bootstrap confidence minBoot = 70 to classify the ASVs, utilizing the MetaZooGene database, to which additional gene fragments, including 18S, have recently been added (https://metazoogene.org/mzgdb/) [70].

After the bioinformatic analysis, we curated the data in different refinement steps for both markers. First, a blank correction was performed, by taking all extraction blanks and PCR negative controls into account. We removed all MOTUs (COI) or ASVs (18S), which were represented with more than 10% of the total reads in the extraction controls from the datasets. All COI sequences were double-checked using BOLDigger [71] and were corrected if a higher taxonomic rank was achieved using BOLDigger or the MOTU was initially assigned to a species known to be distributed outside of the target geographic region. All COI MOTUs assigned with a best identity score less than 0.85 and terrestrial taxa were removed from the dataset. Additionally, all MOTUs assigned to fungi, algae or rotifers were removed from the dataset, since these are likely to result from secondary predation. For the same reason, we kept only ASVs (18S) assigned to Animalia. All ASVs with a bootstrapping value less than 70 at phylum level were removed from the dataset (following [22]). For both markers, ASVs or MOTUs with less than five reads were removed on a sample-per-sample basis. All reads assigned to the predator species were removed for COI. For 18S, which is known to have a lower taxonomic resolution [72,73], we identified reads as potentially representing the predator’s DNA when they were assigned to the order level to which the different predator species belong, in this case: Pleuronectiformes, Gadiformes, Perciformes and Argentiniformes. We removed reads assigned to each of these taxa for all fish samples, to avoid false assignments within the other samples due to tag-switching. Of the remaining MOTUs and ASVs, only those were kept that were plausible to occur in the target geographic region. In the final step, only samples with at least 1000 overall remaining reads were kept.

### 2.5. Data analysis

All data analyses were performed using RStudio (v. 4.3.1) and the packages tidyverse and vegan [74–76]. For data visualization, additionally the package ggpubr was used [77]. After the data refinement, we transformed the reads into relative read abundances (RRA) for further analyses. For each fish species, the percentage of frequency of occurrence (%FOO) and absolute frequency of occurrence (FOO) were calculated for each MOTU and ASV. In order to identify differences in the composition of gelatinous invertebrate taxa in the stomachs of the different fish species, we zoomed into this fraction of the reads and calculated overall RRA considering only the gelatinous invertebrate reads. These overall RRA of the gelatinous invertebrates were then multiplied with the FOO to give a metric of importance of the different species within the gelatinous invertebrate fraction of the reads (following 31). The calculated values were assigned to three categories: less than 100 corresponded to prey taxa of low importance, 101–499 was assigned to be of medium importance, and values greater than 500 were assigned to be of high importance. For three predator species (*G. morhua*, *A. silus* and *S. norvegicus*), we assessed the difference in the prey composition between the different locations around Greenland, which we divided into west, east and south (figure 1). To compare the differences in the prey composition, we applied non-metric multi-dimensional scaling (NMDS) models based on the fourth-root-transformed RRAs for the different predators using Bray–Curtis dissimilarity. We applied PERMANOVAs for the different predator species based on Bray–Curtis dissimilarity of the transformed RRAs. Only one fish species was collected from all three locations (*G. morhua*), for which we performed pairwise comparisons using the pairwise Adonis package in R [78]. The calculated *p*-values were adjusted using the Bonferroni correction [79]. Due to the limited taxonomic resolution of 18S at lower taxonomic ranks [72,73], we only took the family level assignments into account for the analysis.

## 3. Results

### 3.1. Sequencing output

A total output of 20 720 154 reads for COI and 26 892 130 reads for 18S were achieved during the Illumina NovaSeq sequencing run. We removed samples for which less than 1000 reads remained after the curation. Hence, for 27 sequenced stomach content samples of *G. morhua*, we kept 27 and 23 stomach samples for COI and 18S, respectively. Out of 29 stomachs from *A. silus*, we kept 15 samples for COI and 28 samples for 18S. Out of 30 initial stomach samples of *H. platessoides*, we kept 24 and 28 sequence datasets for COI and 18S, respectively. All 12 *M*. *aeglefinus* samples were retained for further analyses, for both markers. For *S. norvegicus*, 15 COI and 16 18S datasets were retained from the 16 sequenced samples. Finally, for the wolffish species, two out of three samples (*A. minor*) and six out of nine samples (*A. denticulatus*) had more than 1000 reads left for both markers after the data curation (electronic supplementary material, table S1).

After the data curation, COI reads ranged from a minimum average of 19 518 reads per stomach for one specimen belonging to *A. denticulatus* to a maximum average of 173 005 reads per stomach for a specimen of *H. platessoides* (electronic supplementary material, table S1). For 18S, reads ranged after the data curation from on average 100 985 reads per stomach for an individual of *H. platessoides* to on average 355 738 reads per stomach for an individual of *A. minor* (electronic supplementary material, table S1). Generally, the reads after the data curation were higher for 18S compared with COI. In some stomachs, reads assigned to the predator species were found to account for the majority of reads, which might give an idea of the fullness of the stomach. The reads assigned to the predator species ranged from 0% of the total reads in the stomachs of *A. minor* to 90.20% of the total reads in the stomachs of *A. silus* for COI. For 18S, predator reads ranged from 0.02% of the total reads in the stomachs of *A. silus* (Argentiniformes) to 36.77% of the total reads in the stomachs of *H. platessoides* (Pleuronectiformes).

### 3.2. Overall prey items of the different fish species in Greenland waters

**Figure 2.**
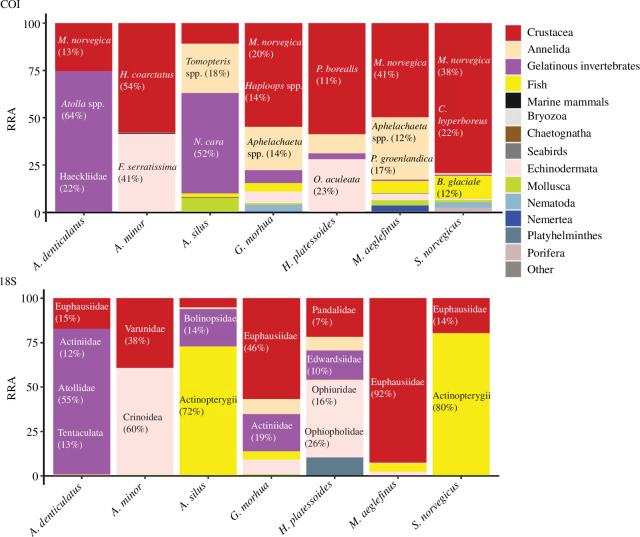
Prey items of the investigated fish species in Greenland waters, for all specimens of each fish species combined. The overall RRAs are displayed for the different predator species. Lower taxonomic levels of the most abundant (in terms of RRA) prey were displayed as text inside the bar plots, with their RRA for both markers.

In the overall stomach content composition of the different fish species (all individual stomachs combined), crustaceans accounted for the majority of reads in both the COI and 18S datasets (see figure 2). However, the crustacean species that dominated varied per fish species, with euphausiids being the main contributor to the crustacean fraction of reads for *A. denticulatus*, *G. morhua*, *M. aeglefinus* and *S. norvegicus*, for both genes. *Meganyctiphanes norvegica* was the most dominant prey item for COI for these species. We have to point out that in this study, we used species-level assignments or higher for COI, while for 18S we used family or higher taxonomic level assignments. For *S. norvegicus* and *M. aeglefinus*, reads assigned to this krill species represented 38% and 41% of the prey reads, respectively. In the 18S dataset, euphausiids accounted for 92% of the prey reads for *M. aeglefinus*, while 18S reads assigned to euphuasiids were the second highest contributor next to fish in the stomachs of *S. norvegicus*. Additionally, for *S. norvegicus* the calanoid copepod *Calanus hyperboreus* accounted for 22% of the overall prey COI reads. Euphausiids accounted for a large proportion of the reads in the stomachs of *G. morhua* of both markers COI (20%) and 18S (46%). In the stomachs of *G. morhua*, we found, apart from euphausiid reads representing the largest fraction for both markers COI (20%) and 18S (46%), reads assigned to amphipods of the genus *Haploops*, accounting for 14% of the prey reads. In the stomach contents of *H. platessoides* and *A. minor,* crustaceans also represented the largest fraction reads for COI (greater than 50%). In the stomachs of *H. platessoides*, the crustacean fraction of the reads could mostly be assigned to the northern shrimp (*P. borealis*) with 11% of the overall prey reads, which in 18S, was represented by 7% of the reads, assigned to Pandalidae. In the stomachs of *A. minor*, the highest read proportion could be assigned to the decapod *Hyas coarctatus* (54% of all COI reads), corresponding to reads assigned to Varunidae in 18S (38%).

Echinoderms accounted for a large fraction of the overall stomach contents of *H. platessoides* and *A. minor*, for both markers. For the contents of *A. minor*, the crinoid species *Florometra serratissima*, contributed with 41% of the COI reads. In 18S, we found crinoids to contribute as prey with 60% of the overall reads. The most dominant echinoderm in the stomachs of *H. platessoides* was the brittle star *Ophiopholis aculeata* (Class: Ophiuroidea), which accounted for 23% of the COI reads. In the 18S results, most echinoderm reads were also assigned to the class Ophiuroidea and the family Ophiopholidae with 16% and 26%, respectively. For *A. silus* and the gadoids *G. morhua* and *M. aeglefinus*, annelids made up significant proportions of the prey reads for COI results, but not for 18S. In the share of annelid reads in the stomachs of *A. silus*, most were assigned to the genus of *Tomopteris*, which accounted for 18% of the prey COI reads. In the stomach contents of *G. morhua* and *M. aeglefinus*, reads assigned to the polychaete *Aphelochaeta* spp. showed the highest annelid contribution with 14% and 12%, respectively. For *M. aeglefinus,* additionally, reads assigned to the polychaete *Phyllodoce groenlandica* accounted for 17% of the COI reads in the stomachs.

In the 18S results, fish represented a major part of the prey items in the stomachs of *A. silus* and *S. norvegicus*, with 72% and 80% of their overall 18S prey reads assigned to Actinopterygii. In contrast, fish contributed less to the overall prey reads in the COI dataset. The only species for which fish was a significant part of its stomach contents was *S. norvegicus* (12% of the total reads assigned to the ice lantern fish, *Benthosema glaciale*). In the stomachs of *A. denticulatus,* reads assigned to GZP species accounted for the largest fraction of overall prey reads after crustaceans for both markers. In the stomachs of *A. denticulatus*, reads assigned to the scyphozoan jellyfish family Atollidae made up 55% of the 18S reads, while the COI dataset had 63% of the reads assigned to *Atolla* sp. Both markers also detected a variety of other GZP species in the stomach content of this species, mainly assigned to ctenophore species. With COI, we found cydippid ctenophores (family Haeckeliidae) to account for 22% of the prey reads, while with 18S, the dominant ctenophores were assigned to the class Tentaculata, accounting for 13% of the overall reads. GZP species also represented a major component of the prey items of *A. silus* in terms of overall RRA. For this species, 52% of the COI reads were assigned to the siphonophore *Nanomia cara* and 14% of the 18S were assigned to the ctenophore family Bolinopsidae. In the stomachs of *G. morhua* and *H. platessoides*, benthic cnidarians were detected. For *G. morhua*, 19% of the 18S reads were assigned to the sea anemone family Actiniidae, while for *H. platessoides,* 10% of the 18S reads were assigned to another sea anemone family, the Edwardsiidae. Benthic cnidarians were also found in the stomachs of *A. denticulatus*, for which reads assigned to the Actiniidae accounted for 12% of the prey reads.

A full list of prey items ingested by the different fish species can be found in electronic supplementary material, table S2.

### 3.3. Spatial variation in the prey composition of Greenland fish species

We analysed the spatial variation in the prey composition for the three fish species *G. morhua*, *A. silus* and *S. norvegicus*, which were sampled at multiple locations (west, east and south Greenland).

**Figure 3.**
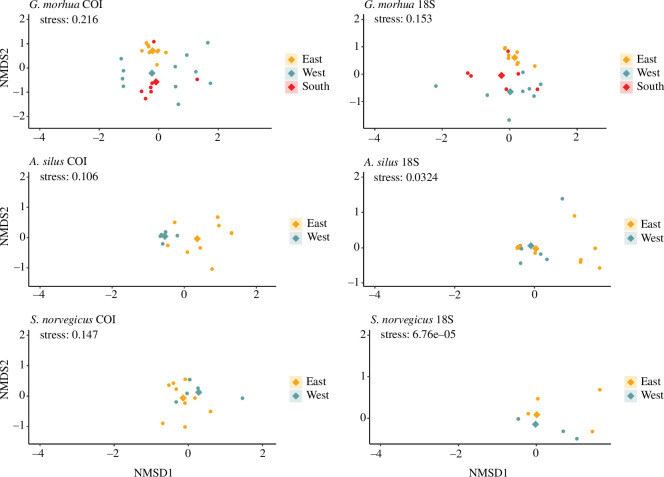
NMDS plots showing the differences in prey spectra for the three fish species *G. morhua*, *A. silus* and *S. norvegicus*, between the different sampling locations in Greenland waters. Dots are colour-coded according to location in Greenland and represent the Bray–Curtis dissimilarity value of the prey composition of one individual. Diamond shapes represent the centroids of the different clusters.

**Figure 4.**
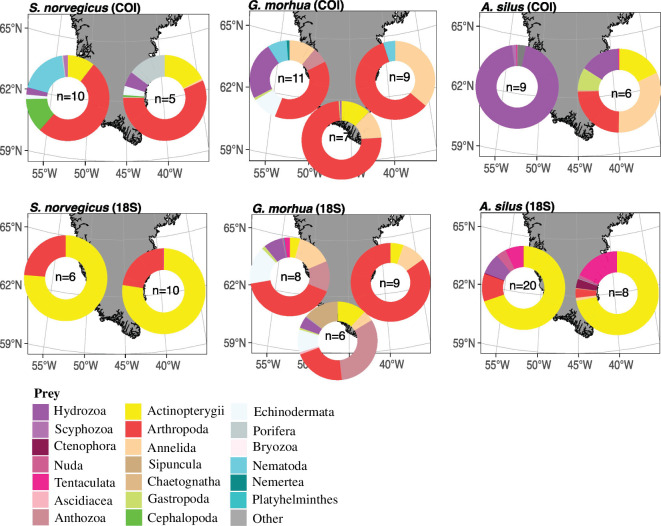
Spatial variation in the prey composition of the different fish species in locations around Greenland based on the COI fragment (upper panels) and the 18S fragment (lower panels).

The PERMANOVA and further pairwise testing showed that the prey composition of *G. morhua* differed significantly between all three locations (east versus west versus south Greenland) in the COI dataset. However, in the 18S dataset, significant differences in the prey composition were only found between south versus east and east versus west. For *A. silus*, we detected significant differences in the prey composition based on the COI dataset, while the PERMANOVA gave no significant differences for the 18S dataset. For *S. norvegicus*, no significant differences were detected in the datasets of both genes. Even though the tested location groups showed no homogeneity in their dispersion for *A. silus* (COI) and *G. morhua* (COI and 18S), the detected differences in the prey composition of *G. morhua* and *A. silus* (only COI) were further supported in the NMDS plots and the prey composition. In the NMDS, we observed distinct clusters and differences in the centroids of the locations for the different predator species (figure 3), which were supported by the prey composition that showed different dominating prey taxa at the different locations (figure 4). For instance, the stomach contents of *G. morhua* were dominated by arthropods at all locations for both markers; however, the dominating crustacean species differed between the three locations. The shrimp species *P. borealis* was most dominant on the west coast (based on COI RRAs) together with the amphipods *Rhachotropis aculeata* and *Syrrhoe crenulata*. On the east side of Greenland, highest COI RRA were assigned to the euphausiid *M. norvegica*, while amphipods of the genus *Haploops* showed the highest COI RRA in the south. The 18S data of *G. morhua* showed similar differences in the crustacean fraction of the prey composition between the three locations, with euphausiids being the most dominant in the east and south, while the west coast was dominated by Caprellidae, Mysidae and Pandalidae with similar contributions in terms of RRA. We found gelatinous invertebrates, mostly Leptothecata hydrozoans and actinarian anthozoans, to contribute largely to the stomach contents of *G. morhua* on the west coast of Greenland, while these were absent in the contents of *G. morhua* on the east coast. For the 18S data, the prey spectra between the west and southern tip were more similar to each other than to that of individuals sampled in the east (figure 4), which was also reflected in the pairwise comparison. The stomach contents of individuals in the south was dominated by actinarian anthozoans, while these only contributed a minor fraction to the prey of individuals sampled in the west and were absent in the stomachs of individuals in the east. Additionally, echinoderms contributed to almost 25% of the RRAs of prey reads in individuals from the west and south, mostly driven by the presence of ophiuroids, while these were also absent as prey in the stomachs of individuals from the east coast. These patterns were also reflected in the NMDS plot, where the highest overlap can be seen between the west and south, while the eastern samples are more similar to each other.

For *A. silus*, significant spatial differences in prey composition were detected between individuals from the west and the east, but only for the COI data, which was also reflected in the NMDS plot. On the western side of Greenland, the prey composition of *A. silus* was dominated by GZP, mainly represented by the siphonophore *N. cara*, while in the east, only 25% of the RRAs detected in the stomach contents were assigned to hydrozoans. Other than GZP, annelids, fish, arthropods and gastropods contributed to the stomach contents of individuals sampled in the east. For the 18S data of *A. silus*, the prey composition did not differ significantly, as both in east and west, the RRAs were dominated by fish (approximately 75%). We only detected a minor spatial variation, with tentaculate ctenophores, assigned to Bolinopsidae, showing higher contributions to the prey composition in the east compared with the west.

For *S. norvegicus*, no significant differences in the prey spectrum were detected for either of the two markers. This is evident in the prey composition revealed by the 18S dataset, where fish (75%) and arthropods (25%) dominated the stomach contents on both sides of Greenland. For COI, the dominant reads in stomach of individuals originating from both sides of Greenland belonged to arthropods, and were assigned to the euphausiid *M. norvegica*, while in the east only, the copepod *C. hyperboreus* contributed almost 30% of the COI reads. However, the remaining reads were assigned to different prey items depending on the location. On the western side of Greenland, the prey consisted of fish (mainly *B. glaciale*), cephalopods and nematodes, with a minor contribution of hydrozoans and scyphozoans. For individuals in the east, we found, besides arthropods, fish, porifera, echinoderms and hydrozoans as prey.

### 3.4. Importance of gelatinous zooplankton and benthic gelatinous invertebrates as prey for Greenland fish species

**Figure 5.**
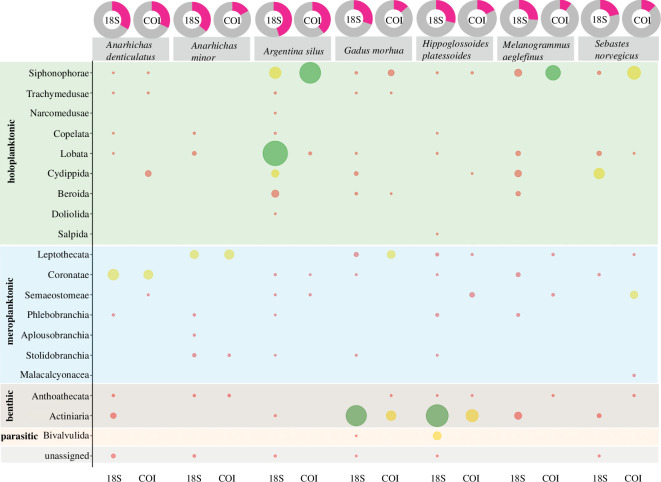
Doughnut charts representing the %FOO of gelatinous invertebrates (pink) in the stomachs of the different fish species for each marker. The bubble plot below represents the importance of the different gelatinous invertebrate species in the stomachs of investigated fish, within the fraction of gelatinous invertebrate species, calculated for each marker by multiplying FOO and gelatinous invertebrate RRAs. Green represents high importance,** **greater than  500; yellow represents medium importance, 101–499; red represents low importance,** **less than 100**.**

The occurrence of predation on gelatinous organisms and their species composition was investigated for all fish species by zooming into this fraction of the reads. Of all fish species investigated, the greater silver smelt (*A. silus*) showed the highest %FOO of GZP species in its stomach contents. We detected reads assigned to GZP in more than half of the 18S samples and in more than a quarter of the COI samples (figure 5). Of all the detected GZP species, holoplanktonic taxa were of high importance in the stomachs, indicated by the applied metric combining FOO and RRAs. Of these, we found siphonophores with high importance for COI and medium importance for 18S, while Lobata (ctenophores) showed high importance for 18S only. Additionally, cyddipids showed medium importance for the 18S reads. After *A. silus*, the two wolffish species, *A. denticulatus* and *A. minor*, showed the highest %FOO of GZP as prey. Gelatinous zooplankton species were detected in a third of the 18S samples for both species and in one-third of the COI samples of *A. denticulatus* and almost a quarter of the COI samples for *A. minor*. Coronatae scyphozoans, represented by *Atolla* spp., showed medium importance in the stomachs of *A. denticulatus*. All other gelatinous groups were mainly found in low importance, with a combination of low FOO and RRA. For *A. minor*, meroplanktonic Leptothecata were detected with both markers, showing medium importance as prey, within the GZP fraction of reads. Overall, even though the FOO for this species were rather high, the RRAs for the different GZP groups were low (figure 5).

Both for *G. morhua* and *H. platessoides*, GZP species were detected with low importance only. However, benthic gelatinous invertebrates, mainly represented by Actinaria, showed high (18S) and medium (COI) importance in the prey composition of *G. morhua* and *H. platessoides*. For *H. platessoides*, parasitic hydrozoans of the order Bivalvulida (parasitic cnidarians) were also found in medium importance in the 18S results. Overall, gelatinous invertebrates were detected in over half the samples for both species for 18S, and in slightly less than a quarter of the samples for the COI data (figure 5). For *M. aeglefinus*, another gadoid species investigated, GZP species were detected in almost a quarter of the samples with 18S, and with COI in less than a quarter of the samples. Holoplanktonic siphonophores were of high importance as prey for this demersal gadoid for COI, but only of low importance for 18S (figure 5). *Sebastes norvegicus* stomachs contained GZP DNA in a quarter of the samples in 18S and less than a quarter in COI. Holoplanktonic GZP, siphonophores (COI) and cydippids (18S), were of medium importance in the prey composition of this species. Additionally, the meroplanktonic order Semaeostomeae, represented by *Cyanea* spp., were of medium importance for COI (figure 5).

### 3.5. Differences in prey species detection between the two markers

We found considerable differences in the detection of prey items based on the markers used, with several prey items being only detected by one of the two markers. For example, reads assigned to the pelagic tunicate groups, Thaliacea and Appendicularia, were detected only with 18S, albeit in small quantities. Ctenophores were detected in higher RRAs (and FOO) with 18S compared with COI. In contrast, cephalopods, priapulids and some parasites were only detected with COI. Furthermore, the fraction of reads assigned to hydrozoans was higher in the COI dataset compared with 18S. Generally, the two markers showed similar patterns in the overall prey composition (figure 2). This was true for the prey composition of *A. minor*, for which both markers showed equal contributions of echinoderms and arthropods. In the case of *A. denticulatus*, similar read proportions of hydrozoans, tentaculate ctenophores and arthropods were identified as prey with both markers (figure 2). For *A. denticulatus*, anthozoans were mainly identified in the 18S data (figure 2). For other species, such as *H. platessoides* and *M. aeglefinus*, larger differences between the markers were visible. For *H. platessoides*, the COI results revealed mainly arthropods, echinoderms and annelids as prey, while the 18S dataset revealed larger contributions of Anthozoa and Platyhelminthes (figure 2). The prey composition of *M. aeglefinus* was dominated by arthropods based on the 18S and COI datasets, but for 18S, a minor contribution of fish species was revealed, while for COI, annelids, nemerteans and echinoderms were detected as additional prey (figure 2).

## 4. Discussion

In this multi-marker metabarcoding study, we provide new information about trophic relationships of seven pelagic and demersal fish species with different life traits and feeding strategies in Greenland waters. For some fish species, this study brings the first insights into their prey spectrum, e.g. for the wolffish (*A. denticulatus*) and the greater silver smelt (*A. silus*). Additionally, we provide insights into the spatial variability in prey composition of Atlantic cod (*G. morhua*), greater silver smelt (*A. silus*) and golden redfish (*S. norvegicus*). We also aimed to disentangle so far unknown predator–prey relationships between fish and GZP as well as to provide evidence for incongruence between different genetic markers to efficiently detect certain prey taxa including GZP.

We provided evidence of GZP predation for all seven fish species investigated, detecting a surprisingly diverse spectrum of gelatinous taxa (59 taxa), including appendicularians, tunicates, ctenophores, hydrozoans and scyphozoans. Such a wide diversity of gelatinous prey taxa has not been documented before (e.g. [54,80,81]). We showed that GZP predation is more common among fish species in Greenland waters than previously assumed, and hence provided evidence to refute the paradigm of GZP being a trophic dead end. Besides GZP, also benthic gelatinous invertebrates were found, e.g. ascidians and sea anemones in the stomachs of Atlantic cod and American plaice, for which they were found to be of high importance. Even though the overall RRAs were rather low for most GZP prey taxa, ranging between 0.1% and 63.7%, FOOs ranged between 1 and 24, indicating that predation on GZP might be more common than previously assumed based on studies using only visual identification methods. As an example, we found ctenophore DNA assigned to the family Mertensiidae in 21 out of 28 stomachs of the greater silver smelt (*A. silus*), while these accounted for only 0.9% of the overall prey reads of this predator. Generally, GZP were found in more than 10% of the samples of all investigated fish species; however, not all GZP species occurred in equal amounts, and fish species appeared to target different species characterized by distinct life-history traits.

### 4.1. General prey composition of the different Greenland fish species

Atlantic cod (*G. morhua*) is generally assumed to be an opportunistic predator feeding on both pelagic and benthic organisms, which was corroborated by our results. Indeed, we were able to identify a high diversity in different prey items (most with rather low RRA and FOO), including benthic organisms like cnidarians and annelids, but also including pelagic crustaceans. The overall cod prey composition appeared to be dominated by crustaceans: particularly euphausiids (*M. norvegica*) and amphipods, belonging to the genus *Haploops,* were abundant in terms of RRA. Other than crustaceans, annelid worms (genus *Aphelochaeta*) accounted for a larger fraction of the COI reads, and sea anemones belonging to the family Actiniidae accounted for a larger fraction of the 18S prey reads. In previous studies, Atlantic cod was found to mainly feed on crustaceans, with the northern shrimp (*P. borealis*) being the most dominant crustacean preyed upon by individuals from northwest Greenland [82–85]. However, in the waters of western Greenland, the diet of cod shows a strong spatial variability [56]. Northern shrimp was found to be dominating in the diet of cod in northwestern Greenland, while in southwestern and east Greenland, euphausiids (*M. norvegica*), were found to be dominating in their stomach contents [55,56]. Conversely, in our study, we found northern shrimp to only account for a small proportion of the overall prey reads (3%) and to be only detected in 6 (COI) and 9 (18S) out of 27 and 23 stomachs, respectively. Other fish accounted for only a small proportion of the overall prey of Atlantic cod, with capelin (*Mallotus villosus*) representing the highest RRA value (2% COI). Capelin was a major part of the diet of Atlantic cod in a past decade (1989–1999), but after that it was no longer assumed to be a major prey due to its decline in local abundances [82,86]. However, in recent studies, capelin was found to provide a large proportion of the diet of Atlantic cod in east Greenland [55,56], which was not supported by our findings.

The stomach contents of haddock (*M. aeglefinus*) were dominated by crustaceans. For 18S, 92% of the reads were assigned to Euphausiacea; for COI, 41% of the reads were assigned to the euphausiid species *M. norvegica,* which might reflect the local abundance of krill in the region. Annelids and other fish species were also part of its prey. In previous studies, crustaceans, echinoderms and fish were found to be dominant prey items of haddock [87]. Stable isotope studies on haddock sampled in southern Norway and the Barents Sea have identified predation on both pelagic and benthic organisms [87,88], which is in line with our findings, showing a predominance of krill in terms of RRAs, but also a large share of annelid DNA. Jiang and Jørgensen [87] identified echinoderms to be dominating in the diet during the last quarter of the year, which overlaps with our sampling period. We did not identify echinoderms to be a major prey of haddock, but the size of the individuals caught could play a role. Jiang and Jørgensen [87] found that small haddock consume less echinoderms and rather feed on crustaceans and annelids. Half of the haddock selected for our study were between 7 and 33 cm long and correspond to these smaller size classes.

For the greater silver smelt (*A. silus*), GZP was the most dominant prey component, in terms of RRA. After GZP, polychaetes assigned to the genus *Tomopteris* (18% of the overall prey reads) dominated for COI, whereas for 18S, fish were the most dominant prey group (72% of the overall prey reads). This dominance of fish as prey in the 18S dataset might be an artefact of the predator removal step, since only few reads were assigned to Argentiniformes, hence, a proportion of the reads assigned to Actinopterygii might still represent the DNA of the predator. We found the majority of stomachs to be empty, with some unidentifiable material. Such a high amount of visibly empty stomachs was also observed for greater silver smelt from other regions. Bergstad *et al*. [89] conducted diet studies in Skagerak and found 52% of the investigated stomachs to be visibly empty. For greater silver smelt from the Rockall Trough, most stomachs were found to be visibly empty [54]. At both locations, a high proportion of stomach contents was unidentifiable [54,89], similar to our findings. By applying DNA metabarcoding, we were able to identify a number of prey items, including GZP, for specimens with visibly empty stomachs. This demonstrates the clear advantage of this method being able to identify highly digested material, compared with traditional stomach content analysis methods. Finally, the diet of greater silver smelt was found to vary between locations in previous studies (e.g. [54,90,91]). In the Rockall Trough, the silver smelt’s diet was dominated by fragments of salps and ctenophores, with also large proportions of content being unidentifiable [54]. On the Scotian Shelf, greater silver smelt mainly fed on pelagic crustaceans (euphausiids and amphipods) [90] similar to east Greenland, where its diet was also dominated by pelagic crustaceans (copepods and euphausiids) [91]. In our study, crustaceans only accounted for a small proportion of the overall prey composition, which is a surprising result, since other studies conducted during the winter months found crustaceans to dominate as prey at other locations. However, these studies reported high numbers of visibly empty stomachs and unidentifiable prey items to account for the largest proportion of the stomach content. We assume that the diet in other regions may also be dominated by GZP, since this group accounted for the largest proportion of the RRA found here.

Our findings on American plaice (*H. platessoides*) were in line with the findings of previous studies, which reported crustaceans as dominant prey. It included, besides the northern shrimp (*P. borealis*), also hyperiid and gammarid amphipods [92,93]. In our study, the largest share of the 18S reads were assigned to brittle stars (Ophiopholidae with 26% and Ophiuridae with 16%), and a major fraction of the COI reads were assigned to the brittle star *O. aculeata* (23%). In the Gulf of Maine, brittle stars also dominated the diet of American plaice [94] and along the East Canadian coast, echinoderms, next to polychaetes and anthozoans, were frequently consumed [92]. Conversely, only a minor share of the diet of North Sea American plaice was represented by echinoderms [93]. Since American plaice is considered to be an opportunistic predator [93,95,96], a spatial and temporal variation in prey composition is expected, as it depends on the available prey field.

The dominant prey of the golden redfish (*S. norvegicus*) consisted of crustaceans, including euphausiids (*M. norvegica*) and copepods (*C. hyperboreus*). These findings are in line with previous redfish dietary studies, in which crustaceans represented the main prey. On the west Greenland continental shelf, these were represented by unidentified copepods and shrimps (Mysidacea), dominating the winter diet [97] and along southwestern Norway, copepods (unidentified and *Calanus finmarchicus*) and hyperiid amphipods (*Themisto* spp.) predominated [98]. As other major prey besides crustaceans, fish, including other redfish, were an important part of the winter diet in west Greenland [97]. We also found fish DNA to occur in high RRA for both 18S and COI compared with other prey items. However, since we removed all reads assigned to the predator species itself, we cannot identify cannibalism or predation on other redfish species in our study. Overall, 40% of the COI reads were assigned to *Sebastes* spp., which was not higher than the amount of predator reads for the other fish species, thus we could not find clear evidence for the predation on other redfish specimens. Besides failing to detect cannibalism, the DNA metabarcoding approach is also limited in detecting secondary predation. Whereas primary predation may be reflected in a higher proportion of reads so that thresholds can be defined to differentiate reads resulting from one or the other [30], it remains challenging to account for this.

The prey composition of the two wolffish species strongly differed: for the spotted wolffish (*A. minor*), dominant prey were crustaceans and echinoderms, whereas these were GZP (mainly ctenophores) and crustaceans for the northern wolffish (*A. denticulatus*). These findings correspond to earlier studies from the Canadian Arctic based on morphological examination [80]. For the spotted wolffish, echinoderms, crustaceans, worms, molluscs and other fish dominated the diet [80]. For the northern wolffish, ctenophores as well as other GZP taxa, crustaceans, echinoderms and fishes were reported to be part of its diet [80]. The prey composition of spotted wolffish investigated in the current study suggests a mix of pelagic and benthic feeding in Greenland waters, as it feeds mainly on benthic crustaceans (*H. coarctatus*) and feather stars (*F. serratissima*), but also calanoid copepods (*C. finmarchicus*) were found in a few stomachs with little contribution to the overall RRA (electronic supplementary material, table S2). Previous studies found evidence for a mixture of benthic and pelagic feeding depending on the size of the specimens, the latter including predation on northern shrimp [99–101]. The prey composition of northern wolffish was dominated by scyphozoans and ctenophores, as well as by the northern shrimp (*P. borealis*), suggesting a predominantly pelagic feeding strategy. Fish were also commonly observed in the northern wolffish diet in earlier studies [99,100,102], but fish predation was not detected here. However, spatial and seasonal variation in feeding can be significant, meaning that snapshots from one area and time point are not unlikely to differ from studies taking place in other regions and during another time of year.

### 4.2. Spatial variance in prey composition of Atlantic cod, greater silver smelt and redfish

The largest spatial differences in prey detected for Atlantic cod (*G. morhua*) were related to the composition of the crustacean prey fraction, which dominated at all locations. Benthic amphipod species *S. crenulata* and *R. aculeata*, as well as caprellid amphipods, together with the northern shrimp and mysids dominated in west Greenland, while the euphausiid *M. norvegica* dominated along the east coast. Similar findings reported the dominance of northern shrimp in cod’s diet on the west coast, while it was almost absent from its diet on the east coast [82]. The authors assumed that the difference in the prey composition of Atlantic cod between west and east Greenland probably reflects the local abundance of prey rather than selective feeding of Atlantic cod [82]. In the south, the prey composition of Atlantic cod was dominated by amphipods of the genus *Haploops* and the euphausiid *M. norvegica*. Additionally, the detection of DNA assigned to fish species, including capelin (*M. villosus*) and sandeel (*Ammodytes dubius*), in the individuals from southern Greenland was striking, as these prey species were so far found in low abundances only [56]. Some studies have pointed out that capelin is generally a more nutritious food for Atlantic cod compared with northern shrimp, and thus is usually favoured if available in sufficient abundances (e.g. [103]), which might be the case for this locality. However, the proportion of reads assigned to fish species are still low compared with the proportions of different crustacean taxa. Hence, a comparison of the spatio-temporal variation in prey composition with the available prey spectrum is needed to draw conclusions on selective feeding.

Based on the COI dataset, the greater silver smelt’s (*A. silus*) prey composition was dominated by the siphonophore *N. cara* on the west coast, while on the east coast, polychaetes (mainly *Tomopteris* and *Aphelochaeta* spp.) and crustaceans (mainly the amphipod *Onisimus nanseni*), mostly contributed as prey. Our results on east Greenland silver smelt corroborates previous findings for this region by Klimpel *et al*. [91], revealing a high contribution of crustaceans (mainly copepods and euphausiids). The prey composition of greater silver smelt from the west Greenland shelf has, to our knowledge, not yet been investigated. However, greater silver smelt is assumed to prey mainly on crustaceans, chaetognaths, ctenophores and other fish species, but previous studies provide no abundance data of these different prey items. In our study, the siphonophore *N. cara* was a major prey on the west side of Greenland, whereas ctenophores were more abundant in the stomach contents of greater silver smelt on the east side, which was not reported in previous studies. Based on the 18S data, dominant prey were fish and euphausiids on both sides of Greenland, this high proportion of fish reads, however, might include predator DNA. No significant differences between the two sampling locations were identified in the 18S dataset, GZP taxa were detected in similar contributions at both locations.

We found no significant spatial difference in the prey composition of golden redfish (*S. norvegicus*). However, despite the dominance of the krill *M. norvegica* as prey on both sides of Greenland, small differences in prey composition were noticeable between east and west Greenland. For instance, the copepod *C. hyperboreus* was only found in high RRAs in the stomachs of individuals from the east coast of Greenland, but absent on the west coast. The latter is in contrast with Pedersen and Riget [97], who found the winter diet of redfish on the west coast of Greenland to be dominated by redfish and copepods. As mentioned before, we cannot differentiate in this study between predator DNA and ingested redfish. However, for 18S, we found fish as prey on both east and west coast in high RRAs, while for COI, the contribution of fish reads was higher on the east coast. These reads were identified as prey and assigned to ice lantern fish (*B. glaciale*) both on east and west Greenland, a prey species which has not yet been reported in previous studies.

### 4.3. The occurrence of gelatinous zooplankton predation in the Greenland fish community

We detected GZP species in the stomachs of all investigated fish species, albeit to different extents. The highest FOO and RRA values for GZP species were detected in the stomachs of greater silver smelt (*A. silus*) and northern wolffish (*A. denticulatus*). GZP (COI and 18S) reads accounted for the majority of the detected prey reads and occurred in almost half to two-thirds of the total reads for the greater silver smelt and northern wolffish, respectively. These two species were known to feed on GZP species in other localities [54,80,99]. For the greater silver smelt, salps and ctenophores were reported as dominant GZP prey [54], while we found the diet of *A. silus* to be dominated by the siphonophore *N. cara* (COI) with only a minor contribution of ctenophores, the latter detected only with 18S. This difference might be caused by the local and seasonal population dynamics of the different GZP species, with their availability as prey determined by their natural population fluctuations or blooms [24]. A seasonal shift in the GZP species preyed upon has been observed in the Atlantic mackerel (*Scomber scombrus*) of the Irish Sea, with a shift from scyphozoans as prey earlier in the year to a diet dominated by smaller hydrozoans [23,24]. In the prey composition of northern wolffish, scyphozoans belonging to the genus *Atolla* played a major role. *Atolla* species are deep-sea scyphozoan jellyfish that are often observed close to the seafloor [104]. The northern wolffish is known to be an epibenthic fish species that is observed in both shallow and deep waters, up to 1700 m depth [80]. In previous studies, ctenophores were documented to dominate as prey for this species, while scyphozoans were only found in low amounts [102]. In our study, ctenophores, the majority of which are assigned to the taxa Haecklidae (COI) and Tentaculata (18S), contributed with 22% and 17%, respectively, of the overall prey reads, but were classified as prey of low importance, while scyphozoans, in particular *Atolla* sp., were of high importance by contributing 64% of the total reads. In Greenland waters, the scyphozoan species *Atolla* sp. and *Periphylla periphylla* dominate the GZP community in terms of wet weight during May and June [52]. Whether their dominance persists till the autumn season is currently not known. The difference in prey composition of northern wolffish revealed here and in previous studies, may find its origin in the local and seasonal fluctuation in GZP composition, since GZP populations generally follow a boom-and-bust cycle and can fluctuate in abundances on various temporal scales [17].

For haddock (*M. aeglefinus*), GZP predation did not play a major role in terms of RRA. However, we detected GZP DNA in almost a quarter of the stomach samples, indicating that GZP are a regular prey for this fish species. Holoplanktonic siphonophores appeared to be of high importance within the fraction of GZP prey, which have not yet been, to our knowledge, reported as a common prey item for haddock. For the golden redfish (*S. norvegicus*), GZP reads were also recovered from almost a quarter of the stomachs, hinting at a frequent predation, despite being detected in low RRAs. GZP predation has previously been reported for redfish in the Irminger Sea, including ctenophores and hydrozoans as prey early in the year (April–May) and most frequently detected in the stomachs of larger individuals [105]. In the Barents Sea, redfish were found to feed year-round on GZP, mostly represented by ctenophores, with a peak in GZP occurrence in the stomachs between October and December [106]. GZP predation appears to be dependent not only on the size of individuals within a species [105], but its occurrence also differed between the different redfish species, with larger amounts of GZP reported from the diet of the Atlantic redfish (*S. mentella*), but an absence of GZP prey was noted for the smallest of the three redfish species, the Norway redfish (*S. viviparus*) [106]. Finally, hydrozoan reads assigned to Leptothecata were found to be prey of medium importance among the GZP species for the spotted wolfish, even though the general contribution of GZP as prey was minor. Predation on ctenophores has previously been reported for the spotted wolffish in the Barents Sea and NW Atlantic [99,102]. These taxa were not playing a major role in the prey composition of the individuals investigated here, but their number was limited to two specimens only. Since the other prey species identified in the stomachs were benthic species, including crabs and brittle stars, the spotted wolffish may have fed on benthic Leptothecata species.

For the American plaice, we found GZP to be of minor importance as prey, whereas in a previous study conducted in the NW Atlantic (Flemish Cap), ctenophores accounted for 15% of its diet [107]. Instead, benthic cnidarians (sea anemones) were found to be of high and medium importance for American plaice among the gelatinous invertebrate species detected. The same was found for Atlantic cod, for which almost a quarter of the samples contained DNA assigned to gelatinous invertebrates, with sea anemones and Leptothecata being of medium importance. In the Barents Sea, Atlantic cod frequently ingested ctenophores, especially in the period between August and December, even when other common prey items were available [81]. In our study, pelagic feeding on ctenophores did not play a major role, whereas benthic cnidarians were prey of higher importance. The absence of feeding on ctenophores could have a seasonal, but also a spatial reason, since GZP communities in the Labrador and Irminger seas are more dominated by scyphozoans in terms of wet weight (e.g. *P. periphylla, Atolla* spp.) [52], whereas in the Barents Sea ctenophores may have become more abundant over the last years due to climate change [81]. A variation in feeding strategy between benthic versus pelagic feeding is commonly observed in Atlantic cod (e.g. [55]) which may explain the difference in the gelatinous prey composition in the current study.

We show that GZP predation is more common and the GZP prey targeted are more diverse than previously assumed for several fish species, hence, providing additional evidence to refute the paradigm of GZP being a trophic dead end. However, it remains unclear whether a frequent ingestion of GZP can account for the energetic demands of these fish species, since the nutritional value of GZP groups is generally assumed to be low. Nonetheless, data on nutritional value only exists for a handful of GZP species. Such studies have pointed out that the energy content of GZP highly differs between different species (e.g. [108]), arguing for a differentiation between taxonomic groups instead of considering GZP as a single group in food-web models. Studies also reported that the amount of essential fatty acids as well as the general energy content are highly dependent on the development of gonads [108,109]. Some predator species were found to specifically target the gonad tissue of GZP species [110]. Generally, when abundances of GZP are higher compared with other zooplankton species, it has been calculated that predators can gain more energy by preying on GZP than on crustaceans [108]. The waters in south Greenland can be dominated by GZP in terms of wet weight in May and June [52]. A comparison of the seasonal GZP composition and abundances is needed to understand whether GZP predation increases in certain periods characteristic of high abundances or blooms. Furthermore, evidence for local or seasonal selective feeding on certain GZP species can only be obtained when comparing the ingested prey with the available zooplankton prey field. Finally, GZP predation is often interpreted to originate from net-feeding, which cannot be excluded, unless applying different capture methods.

### 4.4. Advantages of a multi-marker approach

We applied amplicon-sequencing of two genetic markers, one mitochondrial and one nuclear gene fragment, in order to increase the coverage of detected prey items for the different fish species investigated. Both markers have been previously applied in molecular stomach content analyses, demonstrating their efficiency for detecting metazoan prey, including GZP species (e.g. [22,30,59,111]). The two markers showed an overlap in different prey groups detected, and hence combined, the two datasets reinforce the probability of correct taxonomic assignments. This was the case for several arthropod or echinoderm taxa, as well as for GZP species (e.g. *Atolla*). We also found incongruences in the detection of GZP taxa. For instance, we detected a larger variety of ctenophores with the 18S marker compared with COI, while with COI, a larger diversity of hydrozoans, especially, siphonophores was detected. Salps, doliolids and appendicularians were only detected using 18S. Our study proves that the use of two genetic markers can give a more complete picture of the prey composition, particularly when targeting GZP. However, it has to be noted that the two markers used have different taxonomic resolution in terms of MOTU and ASV assignments [72]. The taxonomic level and number of assignments is dependent on the quality and diversity of the reference database used [72]. For barcoding (Sanger sequencing), a mitochondrial 16S gene fragment was suggested to be more efficient in species delimitation of hydrozoan species than COI [112,113]. Zheng *et al*. [113] pointed out that 16S was more straightforward to be amplified and sequenced for hydrozoan species and that its phylogenetic resolution potential was greater in 16S compared with COI. Finally, the amount of sequences deposited in databases for hydrozoans is higher for 16S than COI, which might lead to better taxonomic assignments for metabarcoding studies as well [113]. For DNA metabarcoding, no 16S marker has yet been designed to target metazoan taxa, but the development of such marker may improve the detection of GZP diversity for future studies. Finally, the most comprehensive insights can be obtained with a combination of temporal snapshot investigations, i.e. stomach content analyses based on morphology and DNA, together with biomarker analyses, for obtaining a longer-term signal on the feeding mode.

## 5. Conclusion

Using multi-marker DNA metabarcoding applied on stomach contents of seven different Greenland fish species, we were able to give new insights into their prey composition for the late autumn period and demonstrated evidence for the importance of GZP in the regional food web. We were able to show that GZP played a role as prey for all investigated fish species, albeit to different extents. Our study shows that within the GZP proportion of the stomach contents, different GZP species with distinct life history traits had a varying degree of importance for the investigated fish species. This proves that the wide diversity of GZP species occupies different roles and positions in the marine food web, and should be considered on a taxon-per-taxon basis rather than generalizing GZP as one single group. Based on DNA, we cannot distinguish whether the fish fed on the benthic polyp stage, the pelagic larvae or the adult pelagic medusae, and it remains challenging to detect cannibalism and secondary predation. Furthermore, our study only provides a temporal snapshot, considering only the prey that was recently ingested and thus, continuous sampling throughout the year combined with observations of the GZP communities are necessary to get a better understanding of the trophic links between fish and GZP. Additionally, we were able to show that the use of multiple genetic markers is beneficial in stomach content studies, to deal with known primer affinities towards specific taxa and to increase taxonomic coverage and precision. Nevertheless, further development of analysing tools specific for multi-marker approaches are necessary to overcome their differences in taxonomic resolution and hence require distinct bioinformatic procedures.

## Data Availability

The raw metabarcoding sequencing data from this project are publicly available at NCBI on the SRA database under the accession number: PRJNA1099445 and the following link: https://www.ncbi.nlm.nih.gov/sra/PRJNA1099445. Supplementary material is available online [114].
